# The Effect of Nonterminal Liquid Crystalline Epoxy Resin Structure and Curing Agents on the Glass Transition of Polymer Networks

**DOI:** 10.3390/polym16060857

**Published:** 2024-03-21

**Authors:** Maciej Kisiel, Beata Mossety-Leszczak

**Affiliations:** Department of Industrial and Materials Chemistry, Faculty of Chemistry, Rzeszow University of Technology, al. Powstańców Warszawy 6, 35-959 Rzeszów, Poland; mossety@prz.edu.pl

**Keywords:** epoxy, liquid crystal, thermoset, curing agent, DSC, polymer network, polarized optical microscopy, glass transition, vitrification

## Abstract

Modern science and technology demand a low glass transition temperature, yet one tailored to specific thermoset needs and specific to individual hardener applications. Two novel, nonterminal liquid crystalline epoxy resins (LCER) were synthesised, with their structures characterized via nuclear magnetic resonance (NMR), mass spectrometry (MS), and elemental analysis. Their liquid crystalline nature and thermal properties were determined using polarized optical microscopy (POM), thermogravimetric analysis (TGA), and differential scanning calorimetry (DSC). A set of seven aromatic amines serving as curing agents was used to perform curing in fourteen different systems in order to assess the glass transition temperature (T_g_) of the obtained polymer networks using DSC. The liquid crystalline elastomers were obtained with vitrification occurring in a low temperature range (−10–40 °C), with a more predictable outcome for amines with two aromatic rings in the structure than with one. Moreover, the resin with a core consisting of four aromatic rings produces networks with higher T_g_ than the three-aromatic resin. The use of nonterminal LCER allowed the lowering of the glass transition temperature of the polymers to more than 70 °C compared to a terminal analogue. This brings new possibilities of designing highly elastic yet cured polymers with potential for use in smart applications due to the LC nature of the resin.

## 1. Introduction

Epoxy resins are without a doubt one of the most popular and important thermosetting materials. Studied thoroughly for many years, they have been the subject of some comprehensive review articles [1,2] underlining their broad use and valuable properties, such as chemical resistance, thermal stability, and good mechanical endurance. However, some aspects of epoxy networks needed enhancement, which was possible to achieve with modifications and the addition of fillers [1,3,4]. This allowed the glass transition temperature (T_g_) to be shifted, usually to a higher range; the reinforcement of the matrix to withstand greater mechanical loads; manipulation with conductivity; and importantly a reduction in the cost of production. It has to be noted, however, that modern-day chemists prefer to abandon the use of fillers due to their extremely difficult recycling processes, as the most difficult aspect is probably the proper extraction of fillers from the matrix [5]. Advancement in this matter brought about the invention of liquid crystalline epoxy resins (LCER), which use the liquid crystal (LC) nature of these compounds to facilitate an even greater enhancement of properties than that induced by the addition of fillers. Moreover, the introduction of LCER allowed the design of networks with smart properties, as liquid crystallinity can be induced by facilitating shape memory and an anisotropic distribution of properties like mechanical, electrical, and optical ones. This facet became exhaustively interesting for science and resulted in numerous studies that are now summarised in recent review articles [6].

Even with the flexibility of the possibilities provided by LCERs, sometimes problems with designing a polymer with a proper (and usually low) glass transition temperature (T_g_) can emerge. This can be solved by the use of a precisely selected curing agent, which has been the subject of investigation for many years now. For example, Thakur et al. studied imidoamines and their effect on epoxy thermoset properties [7], and Wang’s and Tong’s groups used biphenyl-based curing agents to assess curing behaviour [8,9]. Regarding a subject that is especially important in the case of LCERs, Carfagna and his team, the great pioneers in this field, found that both the level of curing and the physical properties of the hardener are strongly affected by the nature of the hardener [10], so it is extremely important to investigate a broad selection of curing agents and compare their effects on the network. The importance of this matter has become even greater since LCERs became the foundation for manufacturing high-end materials for 5G antennas [11], tunable actuators with reprocessibility [12], microrobotics and micromachinery [13], highly conductive polymers [14], vitrimers [15], optical devices [16,17], and high-impact-strength polymers [18], as well as for providing a property-enhancing factor in powder coatings [19].

During our previous research, we followed a traditional method of LCER synthesis, placing the reactive oxirane group at the end of a hydrocarbon elastic spacer [20,21], with success, resulting in networks with a T_g_ at around 70–80 °C, but we struggled to lower the vitrification temperature. This led to substantial innovation in the field and the invention of, according to the best of our knowledge, previously undescribed LCERs with oxirane groups in the middle of the spacer. This was possible due to exploitation of oleic acid in the course of synthesis, which also gives great green advantages. The initial results were more than promising [22,23] regarding the electrical, structural, and thermal properties of the network. In the context of this article, the achieved shift in T_g_ from 80 °C to the room temperature region is the worthiest of emphasis, but there are also other interesting features worth noting, like the detection of numerous dielectric relaxation processes or the possibility of achieving molecular order. These affordances are not, however, crucial in the case of this particular paper. Following this notion, we synthesised two nonterminal LCERs with a three- and four-aromatic mesogenic core and an oleic-acid-based spacer and investigated the curing process as well as the T_g_ values of the produced networks. The curing process was performed by using seven aromatic diamines as hardeners.

## 2. Materials and Methods

### 2.1. Materials

The following chemical compounds were used:

- 4,4′-dihydroxybiphenyl, Glentham Life Sciences, Corsham, UK;
- Hydroquinone, Fluka, Buchs, Switzerland;
- DCC; *N*,*N*′-dicyclohexylcarbodiimide, Sigma Aldrich, Steinheim, Germany;
- *p*-hydroxybenzoic acid, Sigma Aldrich, Steinheim, Germany;
- *p*-TSA; *p*-toluenesulfonic acid, Sigma Aldrich, Steinheim, Germany;
- *m*-CPBA; *m*-chloroperbenzoic acid, Sigma Aldrich, Steinheim, Germany;
- oleic acid, Fischer Chemical, Loughborough, UK;
- ethanol, methanol, ethyl acetate, dichloromethane and acetone, Chempur, Piekary Śląskie, Poland;
- decalin, Fluka, Buchs, Switzerland;
- *o*-PDA, *o*-phenylenediamine, Thermo Scientific; Waltham, MA, USA;
- *m*-PDA; *m*-phenylenediamine, Sigma Aldrich, Steinheim, Germany;
- *p*-PDA; *p*-phenylenediamine, Merck, Darmstadt, Germany;
- 3,3′-DDM; 3,3′-diaminodiphenylmethane, Sigma Aldrich, Steinheim, Germany;
- 4,4′-DDM; 4,4′-diaminodiphenylmethane, Sigma Aldrich, Steinheim, Germany;
- 3,4′-ODA; 3,4′-oxydianiline, Angene Chemical, Telangana, India;
- 4,4′-ODA; 4,4′-oxydianiline, Angene Chemical, Telangana, India.

### 2.2. Methods

#### 2.2.1. Synthesis of Nonterminal LCERs

**Figure 1:**
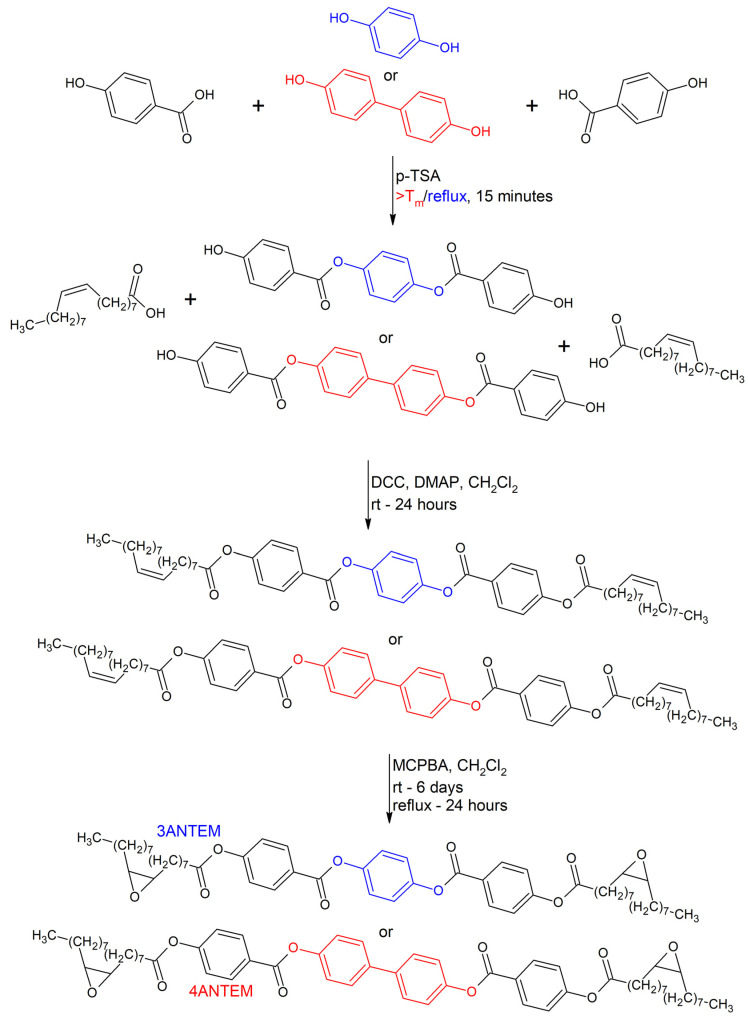
Synthesis scheme for 3ANTEM and 4ANTEM.

The synthesis scheme is presented in Figure 1.

In the first step, stoichiometric amounts of 4,4′-dihydroxybiphenyl (183.2 mmol, 34.11 g) or hydroquinone (183.2 mmol, 20.17 g) and *p*-hydroxybenzoic acid (366.4 mmol, 50.61 g), as well as *p*-toluenesulfonic (0.002 mol, 0.34 g), which was used as a catalyst, were mixed without any solvent in the case of 4,4′-dihydroxybiphenyl and with decalin (30 mL) in the case of hydroquinone in a round-bottom four-neck flask equipped with a nitrogen gas inlet. The mixture was vigorously stirred with a mechanical stirrer and heated up to 290 °C in the case of 4,4′-dihydroxybiphenyl or 225 °C in the case of hydroquinone. After producing these conditions, the process was continued for about 15 min. The crude brown products were cooled and denoted as MEZO III (4,4′-dihydroxybiphenyl) and MEZO II (hydroquinone). MEZO III was ground in a mortar and mixed in boiling ethanol for purification. The mixture was filtered under vacuum and excessively rinsed with hot ethanol until it became white. Afterwards, the white/grey product was dried (63% yield). MEZO II was ground in a mortar and mixed in boiling methanol, vacuum-filtered, rinsed with hot methanol, and dried. A pure, white-beige product was obtained with a 76% yield.

MEZO III: ^1^H-NMR (DMSO), δ (ppm): 10.5 (s, 2H); 8.0 (d, 4H); 7.8 (d, 4H); 7.3 (d, 4H); 6.95 (d, 4H). Elemental analysis calculated (%) for C 73.23 found 73.78, calculated (%); for H 4.25 found 4.24.

MEZO II: ^1^H-NMR (DMSO), δ (ppm): 10.5 (s, 2H); 8.0 (d, 4H); 7.32 (d, 4H); 6.94 (d, 4H). Elemental analysis calculated (%) for C 68.57; found 70.33. Calculated (%) for H 4.03; found 4.14.

In the second step, MEZO III (10 mmol, 4.26 g), oleic acid (20 mmol, 5.64 g), (10% excess) DCC (22 mmol, 4.54 g), DMAP (1 mmol, 0.12 g) (used as a catalyst), and dichloromethane (100 mL) were placed in a three-neck round-bottom flask and stirred at 20 °C for 24 h. The mixture was vacuum-filtered, and the solvent was removed through the use of an evaporator. The crude product was mixed for 15 min in boiling methanol (100 mL), filtered, and dried. A total of 0.007 mol of white solid was obtained with a 73% yield. The product was named 4ANTVM (four-aromatic nonterminal vinyl monomer). The second step of the synthesis using MEZO II (10 mmol, 3.50 g) was conducted with ethyl acetate (100 mL) in place of methanol during product purification (product was fully dissolved and recrystalized), with a similar yield (70%) of white solid named 3ANTVM (three-aromatic nonterminal vinyl monomer). 

4ANTVM: ^1^H-NMR (CDCl_3_), δ (ppm): 8.26 (d, 4H); 7.65 (d, 4H); 7.3 (m, 4H); 7.25 (d, 4H); 5.4 (m, 4H); 2.6 (t, 4H); 2.05 (q, 4H); 1.77 (m, 4H), 1.37 (m, 44H); 0.9 (t, 6H). Elemental analysis calculated (%) for C 77.95; found 78.46. Calculated (%) for H 8.65; found 8.66.

3ANTVM: ^1^H-NMR (CDCl_3_), δ (ppm): 8.24 (d, 4H); 7.28 (d, 4H); 7.25 (d, 4H); 5.45 (m, 4H); 2.6 (t, 4H); 2.07 (q, 4H); 1.75 (m, 4H), 1.35 (m, 44H); 0.9 (t, 6H). Elemental analysis calculated (%) for C 76.50; found 76.57. Calculated (%) for H 8.94; found 8.77.

In the third step of the synthesis, 4ARNTM (10 mmol, 9.55 g), *m*-CPBA (27 mmol, 4.66 g)—with 35% excess assessed based on the assumption that the substrate content was 65% due to its decomposition during storage, so the practical weight of the substrate was 7.17 g—and dichloromethane (100 mL) were placed in a three-neck round-bottom flask, stirred with a magnetic stirrer for six days at 25 °C, and then subjected to reflux (40 °C) conditions for the last 24 h. As *m*-CPBA should be used in excess in order to allow the full oxidation of C=C aliphatic bonds, there was no need for a precise determination of its content. Then, the mixture was cooled and vacuum-filtered, and the filtrate was subsequently washed in a three-step process with 5% Na_2_SO_3_ solution (80 mL), 5% NaHCO_3_ solution (80 mL), and saturated sodium chloride solution (80 mL). After each washing step, the organic layer was separated. After that, the organic liquid was dried with MgSO_4_, and then filtration was performed. The solvent was removed using a rotary evaporator. The crude product was purified by mixing it with boiling ethyl acetate (150 mL), followed by cooling and filtering. After that, the purification procedure was repeated with methanol (150 mL). A total of 0.008 mol of white solid, dubbed 4ANTEM (four-aromatic nonterminal epoxy monomer), was obtained (80% yield). Oxidation of 3ANTVM (10 mmol, 8.79 g) was conducted with smaller amounts of solvents for final purification; methanol (100 mL) and ethyl acetate (75 mL) were used, with the final white solid being named 3ANTEM (three-aromatic nonterminal epoxy monomer), providing a yield of 67%. 

4ANTEM: ^1^H-NMR (CDCl_3_), δ (ppm): 8.35 (d, 4H); 7.75 (d, 4H); 7.35 (m, 4H); 7.25 (d, 4H); 3.1 (m, 4H); 2.75 (t, 4H); 2.00 (q, 4H); 1.5 (m, 48H); 0.9 (t, 6H). MS (^109^AgLGN LDI-ToF) *m*/*z*: calculated for [M-^109^Ag]^+^ = 1095.4114; found 1095.5034. Elemental analysis calculated (%) for C 75.43; found 74.42. Calculated (%) for H 8.37; found 8.18.

3ANTEM: ^1^H-NMR (CDCl_3_), δ (ppm): 8.30 (d, 4H); 7.30 (d, 4H); 7.25 (d, 4H); 3.05 (m, 4H); 2.62 (t, 4H); 2.07 (q, 4H); 1.5 (m, 48H); 0.9 (t, 6H). MS (^109^AgLGN LDI-ToF) *m*/*z*: calculated for [M-^109^Ag] ^+^ = 1019.4638 found 1019.4545. Elemental analysis calculated (%) for C 73.82; found 73.62. Calculated (%) for H 8.63; found 8.51.

#### 2.2.2. Preparation of the Curing Mixture

Thermosetting samples were prepared by adding stoichiometric amounts of 3ANTEM or 4ANTEM and one of the hardeners (in a 2:1 molar ratio). The substrates were weighed in a small bottle, mixed with 15 mL of acetone, and stirred for 2 h. Then, the solvent was evaporated at room temperature until reaching a constant mass, and the mixture was stored in a fridge before curing. Each mixture contained approx. 500 mg of epoxy. This procedure was adopted from our previous research concerning these resins [22,23].

#### 2.2.3. ^1^H-NMR Analysis

Proton nuclear magnetic resonance (NMR) spectra were obtained using a Bruker Avance II Plus spectrometer operating at 500.13 MHz under a magnetic field of 11.7 T. Spectra were obtained with standard instrument software (Topspin 1.3) and pulse sequences (zg30) at a probe temperature of 25 °C. The acquisition parameters for proton NMR experiments were as follows: acquisition time—3.27 s, spectral width—4000–6000 Hz, nutation angle—30°, relaxation delay—1 s, and number of data points—32 K. A 30-degree single-pulse sequence was used for FID accumulation. For all samples studied in this report, a small piece (3 to 5 mg) was dissolved in 0.6 mL of CDCl_3_ using 5 mm NMR tubes. Chemical shifts are expressed in ppm downfield from tetramethylsilane (TMS) as an internal reference. Deuterated solvent with deuterium isotope enrichments of 99.6% were purchased from ARMAR Chemicals, Leipzig, Germany.

#### 2.2.4. DSC Analysis

The DSC analyses were performed with the Mettler Toledo DSC822^e^ apparatus in a nitrogen atmosphere with a nitrogen gas flow of 60 mL/min. The heating rates were 5 °C/min for substrate analysis and curing and 10 °C/min for post-curing determination of T_g_. The temperature range of the measurements was individually selected for each thermosetting system and is presented on every thermogram. The samples’ weight was about 10–15 mg. To determine the stress built up during curing and facilitate the post-curing process in order to achieve maximum possible conversion, two post-curing heating cycles were applied, and the T_g_ value was determined via the analysis of the second one.

#### 2.2.5. POM Analysis

Polarised optical microscopy analyses were performed using a Lab40 microscope (Opta Tech, Warsaw, Poland) equipped with crossed polarisers and an MI20 Opta Tech digital camera combined with a Linkam LTS420 heating stage. During the observations, a magnification of 500× was used. The heating rate was 5 °C/min.

#### 2.2.6. Elemental Analysis

Elemental analysis for C, H, and N was carried out with a Carlo-Erba EA 1108 analyser produced by Thermo Fisher Scientific Inc., Waltham, MA, USA.

#### 2.2.7. Mass Spectrometry Analysis

LDI-ToF mass spectrometry experiments were performed in reflectron mode using a Bruker Autoflex Speed time-of-flight mass spectrometer equipped with a SmartBeam II laser (355 nm). Laser impulse energy was approx. 90–140 μJ, and the laser repetition rate was 1000 Hz. The total number of laser shots was 12,000 for each spot divided into packs of 2000 shots per one measurement point. At each point, 2000 laser shots were made with the default random walk applied (random points with 50 laser shots). The measurement range was *m*/*z* 500–2000. Suppression was turned on typically for ions with an *m*/*z* lower than 500. The reflector voltages used were 21 kV (the first) and 9.55 kV (the second). The data were calibrated and analysed with FlexAnalysis (version 3.3) using a centroid calibration model. Mass calibration (enhanced cubic calibration based on 4–6 calibration points) was performed using external standards (Bruker Peptide Calibration Standard). A drop of 0.5 μL was placed on a ^109^AgLGN plate.

#### 2.2.8. TGA Analysis

Thermal analyses of the prepared samples were carried out in nitrogen atmosphere with flow of 50 mL/min in the 25–700 °C temperature range. The heating rate was 10 °C/min. The analyses were performed using TGA/DSC1 produced by Mettler Toledo, Columbus, OH, USA.

## 3. Results and Discussion

### 3.1. Structural Analysis of the Synthesis Products

**Figure 2:**
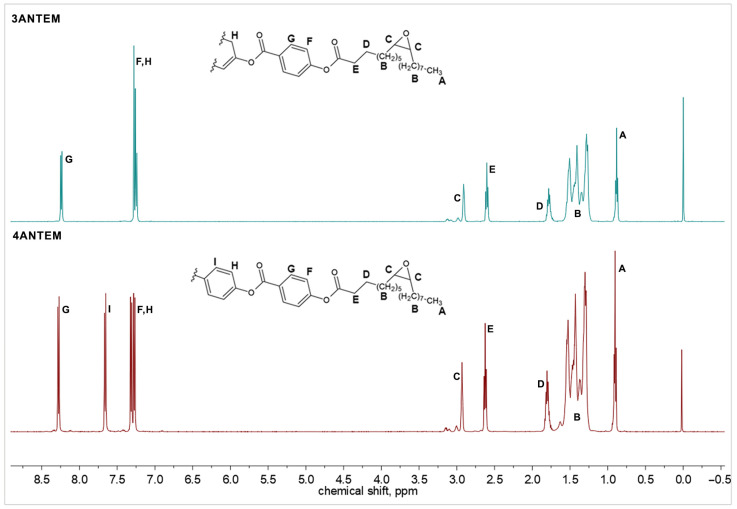
^1^H-NMR spectra of synthesised nonterminal LCERs.

^1^H-NMR spectra for the final products were obtained and are presented in Figure 2.

As visible above, the analysis of the attached spectra provided confirmation of the successful course of the synthesis. All signals were attributed to the corresponding protons of the molecules. Each hydrogen atom from the resin structures is denoted by a distinct letter of the alphabet as well as a resonance signal generated as a sign of the presence of this hydrogen atom, as presented in Figure 2. The calculated integrals also prove the desired molecular structure and allowed us to use the products in the curing process. There are visible residual peaks around 3 ppm that were attributed to oleic acid homologues; the substrate used had 70% purity, and there was a possibility of the presence of similar compounds in the mixture. Oxidation of those compounds could yield a very low, yet visible, number of epoxy groups with an unidentical magnetic neighbourhood.

**Figure 3:**
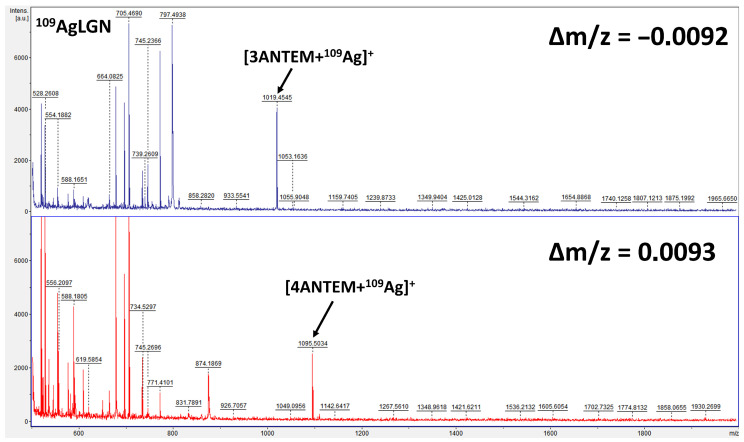
Mass spectra of synthesised nonterminal LCERs.

The MS spectra obtained are presented in Figure 3. The information provided by these experiments clearly confirms the successful course of synthesis because the obtained molecular ions are characterized by proper monoisotopic masses, which differ in comparison to the calculated, theoretical ones by 0.0092 *m*/*z* for 3ANTEM and 0.0093 *m*/*z* for 4ANTEM.

The elemental analysis, with the results placed below the synthetic procedure, also confirmed that the desired product was obtained.

### 3.2. Thermal Characteristics of Resins and Hardeners

Thermal properties have a great influence on the curing process, so it is crucial to investigate them and determine all the temperatures of phase changes, especially melting.

**Figure 4:**
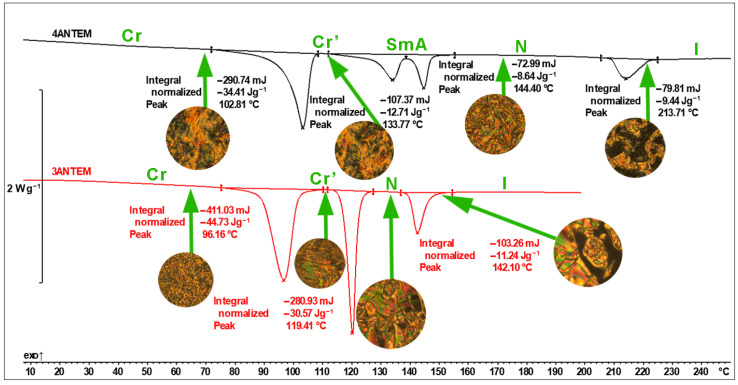
DSC thermal curves and POM microphotographs of synthesised LCERs (Cr—crystalline phase 1; Cr’—crystalline phase 2; SmA—smectic A phase; N—nematic phase; I—isotropic liquid).

In the case of LC thermotropic compounds, DSC thermograms present more than one peak, and additional transitions are also correlated with polymorphic or LC-related transitions. As the DSC technique alone is not sufficient to confirm liquid crystallinity, POM observations of the resins were also made, and the results are presented and analysed in Figure 4 and Table 1.

The data obtained from the first heating runs for both monomers confirm the LC nature of the synthesised resins, as determined through the analysis of POM microphotographs. These photographs were taken above the temperatures corresponding to phase transitions (marked with green arrows in the figure) detected with the use of DSC. Only the first heating run is presented because of the necessity of presenting the same state of compounds that we analysed during curing to avoid mistakes. The differences between the two photographs taken at the lowest temperature for each sample are very subtle. Polarised optical microscopy is often used to distinguish polymorphic forms of crystalline compounds, but in this particular case, they are not clearly noticeable, especially in the observation of 4ANTEM. In the case of 3ANTEM, there are a few more visible changes, which indicate the detection of polymorphism. The second transition has been described as the formation of the mesophase. In the case of 4ANTEM, the process is more complex and consists of two subprocesses (two DSC peaks), but due to their overlapping nature, only the final stage is presented. It seems that the additional, intermediate LC phase could be smectic A [23,24]. The texture presented in Figure 4 is characteristic of nematic liquid crystal [24]. The last pictures for both resins were taken just above the last DSC peak temperature (214 and 142 °C) and show isotropisation. The dark regions with crossed polarisers captured during the POM observations are proof of isotropic, not birefringent, samples. Hence, the birefringent mesophase undergoes a final transition from a liquid crystal to an isotropic liquid. DSC/POM analyses allowed us to conclude that we synthesised thermotropic LCERs with a stable nematic phase and with a smectic intermediate phase in the case of 4ANTEM.

**Figure 5:**
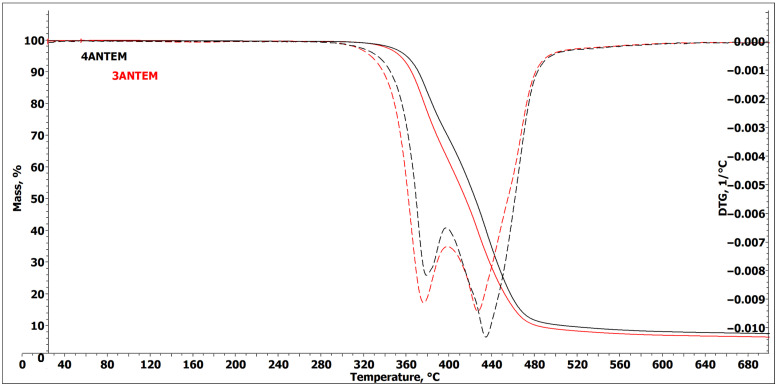
TGA (solid line) and DTG (dotted line) curves of investigated nonterminal LCERs.

LCERs were also subjected to TGA analysis to assess if they would be thermally stable in the curing temperature range. The results are presented in Figure 5.

The thermogravimetry experiments showed similar behaviour for both resins, with slightly better thermal stability for the four-aromatic resin, as was expected. The diagnostic 5% mass-loss temperatures are 325 °C for 3ANTEM and 330 °C for 4ANTEM, so they were stable under the curing conditions. They are, however, the first signs of the thermal degradation of monomers over 300 °C, but when curing reaches this temperature, there are no unreacted substrates in the mixture, and the epoxy network gains stability upon formation. Hence, the thermal degradation of resins during hardening is even less probable. Over 330 °C, intense, two-step thermal degradation starts. The first step is likely related to the degradation of hydrocarbon chains, and the second is related to the destruction of aromatic, stable, rings. This degradation, however, does not influence the curing process.

**Figure 6:**
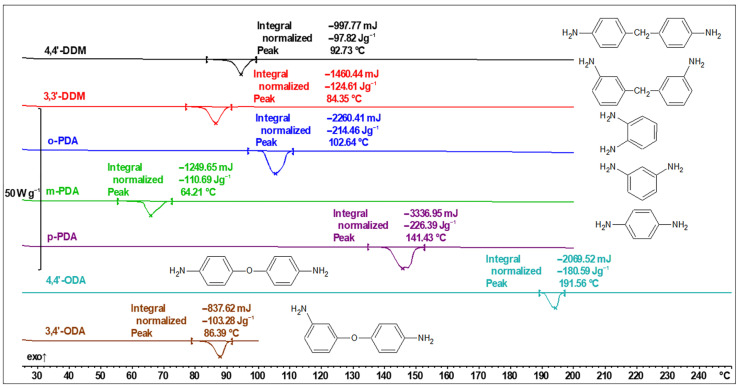
DSC thermal curves and structures of investigated curing agents.

The structure of hardeners as well as their DSC curves are presented in Figure 6.

As presented above, the thermal characteristics are different from one agent to another, sometimes even drastically in the collection of isomers (86 °C vs. 192 °C in case of 3,4′-ODA and 4,4′-ODA). The detected melting temperatures of the curing agents alone are of less importance than their influence on the curing process.

### 3.3. Analysis of the Curing Process

**Figure 7:**
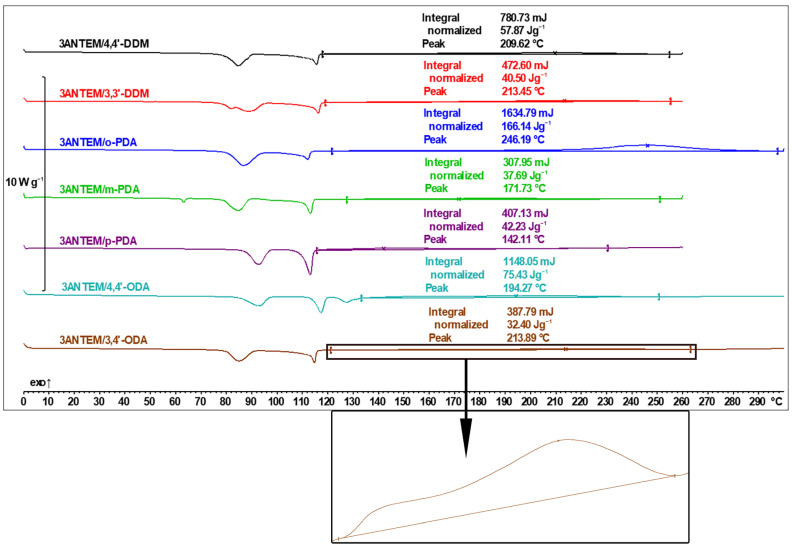
DSC thermal curves of the 3ANTEM curing process.

The curing process was performed with the use of the DSC method, and, for the 3ANTEM resin, the results are presented in Figure 7.

For all the samples, each transition is related to those presented in Figure 4 and Figure 6. If endothermic peaks are present, they are a representation of processes identified via the POM/DSC analysis of the monomer described in Table 1 or the melting of the hardener. Phase changes of the curing agents (3,3′-DDM, *m*-PDA, and 4,4′-ODA) are visible only if this transition does not fully overlap with monomer-related peaks with higher intensity. Above the LC formation temperature, there are wide, exothermic curing peaks, which, for better clarity of presentation, are magnified in the case of the use of the 3,4′-ODA curing agent. 

The DSC data show that in the case of the investigated resin, the curing process has low intensity and gradually progresses with temperature. This is probably due to the monomer structure: the epoxy group in the middle of an aliphatic spacer generates great steric hindrance, thus hindering its connection with the hardeners’ functional groups, even if their molecules are small. The size of the hardener molecule is nonetheless important, which is especially visible in the case of *o*-PDA, where the curing enthalpy is more than two times higher than the second highest (4,4′-ODA) and around five times higher than the lowest (3,4′-ODA). The analysis of oxydianiline isomers brings attention to the possible importance of the symmetry of the hardener structure in facilitating curing reactions. It is possible that the curing is also constrained by the increasing viscosity of the reacting mixture and the rate of curing spikes after exceeding some temperature (viscosity) threshold, which is also especially visible in the case of *o*-PDA. Besides the carefully evaluated exothermal curing peak, there are visible processes associated with pure monomers, which, to ensure the clarity of the figure, were not evaluated mathematically. There are, however, phenomena worth noting: an expected shift in phase transition temperatures to lower values after mixing both substrates (especially visible in the case of 4,4′-ODA) and the distinct deformation of the transition of resin to the LC state.

**Figure 8:**
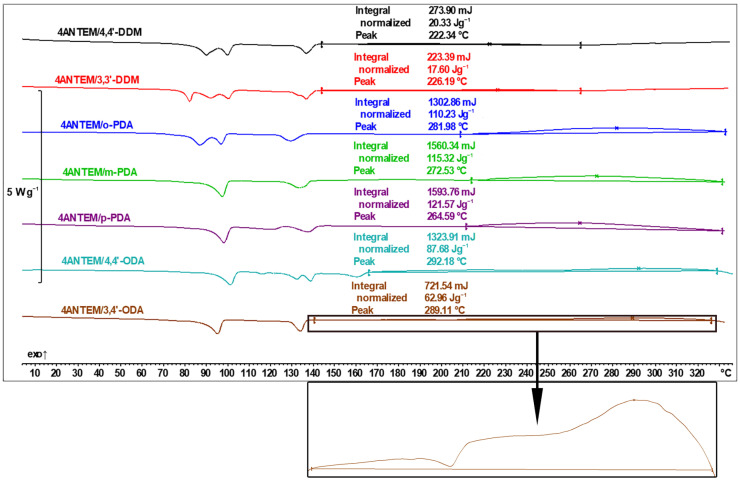
DSC thermal curves of the 4ANTEM curing process.

DSC investigations were also performed for 4ANTEM, and the results are given in Figure 8. Each transition can be identified similarly to those in Figure 7.

The results for the 4ANTEM resin are consistent with the findings for 3ANTEM. The incorporation of four aromatic rings in the monomer structure allowed us to better distinguish the effect of viscosity on network formation. In the liquid crystalline state, below 220 °C, only DDM amines are able to conduct curing with higher intensity. For all other amines, there is a clear increase in heat generation (the rate of reaction) when the resin transitions into an isotropic state, which is especially visible upon magnification (4ANTEM/3,4′-ODA, brown bottom curve). As liquid crystal, due to its ordered nature, always has higher viscosity than an isotropic liquid created from it, the explanation of detected phenomena seems to be clear. Also, the rotational constraint characteristic of LC compounds makes it harder for the hardener molecules to come into proximity with epoxy groups because of the alignment of mesogens next to each other. Characteristic for this resin is also the fact that if curing starts at a lower temperature (as with DDM amines), its extent seems to be lower, which is evidenced by the fact that those systems have the lowest curing enthalpy. A bigger core structure than that in the case of 3ANTEM seems to allow better infiltration of curing agent molecules between resin scaffolds, resulting in a larger amount of generated heat due to the effect of greater conversion.

For the temperature region below the start of the curing, all observations are consistent with the 3ANTEM analysis.

### 3.4. The Effect of Hardener Type and Resin Structure on the T_g_ of the Polymer Network

**Figure 9:**
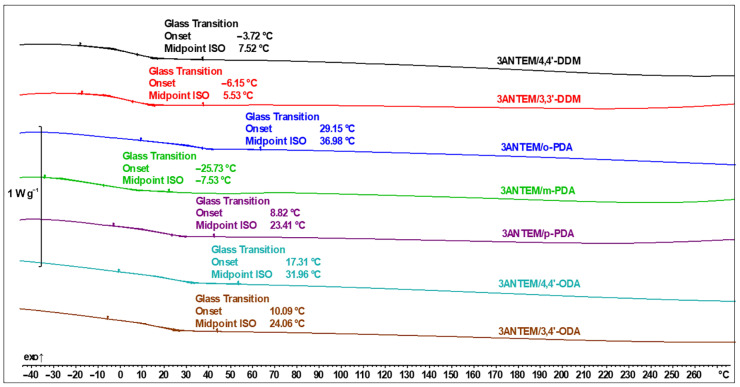
DSC thermal curves with determination of T_g_ of 3ANTEM-based polymer networks.

**Figure 10:**
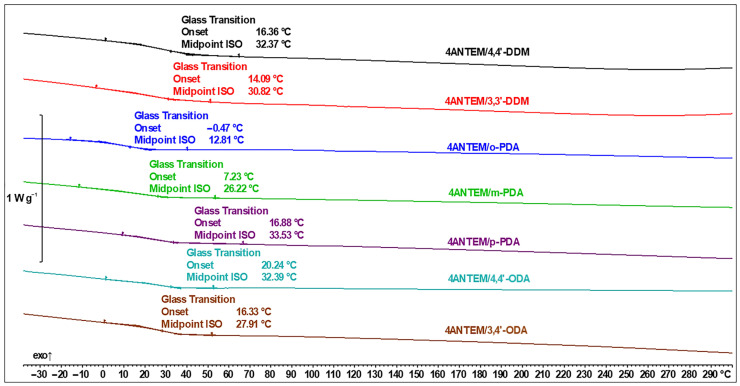
DSC thermal curves with determination of T_g_ of 4ANTEM-based polymer networks.

After performing the curing process, the samples were subjected to heating runs in order to detect their T_g_ values and determine if they had been cured to the maximum possible extent. There are no peaks in the DSC thermograms presented in Figure 9 and Figure 10 that can be correlated with either curing or unreacted monomers, so the epoxy–amine reaction can be described as having been completed.

Our first glance at the results yielded the satisfactory conclusion that using nonterminal LCERs allowed for the achievement of a significantly lower T_g_ than that in systems using the same mesogenic core but with spacers equipped with epoxy groups at the ends of hydrocarbon chains, and this shift could amount to over 70 °C when the same curing agents were used [21]. For better clarity of the analysis, all important data are collected in Table 2.

The dataset presented in Table 2 allows us to draw significant conclusions. First of all, as mentioned before, the quest of achieving low-T_g_ liquid crystalline polymer networks has been successful. Secondly, in the case of sophisticated materials like liquid crystalline epoxy resins also complicated by the introduction of innovative nonterminal epoxy groups, some difficulties with predictions about the even broader use of hardeners have emerged.

By splitting the whole dataset into smaller subsections, it can be concluded that only oxydianilines (ODAs) act in a fully predictable manner. When discussing these samples, we concluded that the introduction of the fourth aromatic ring in 4ANTEM caused a rise in the T_g_ of the polymers as a result of the stiffening of their molecular structures. This is completely logical, because vitrification, as a process describing the molecular motion of polymer chains, is more restricted and energy demanding, with bigger and stiffer four-aromatic than three-aromatic cores of the network. Also, by comparing the extent of the cure measured by the heat of the reaction, one can conclude that bigger resin molecules facilitate hardening in this case. This can be explained by the easier penetration of hardener molecules between larger resin scaffolds, because it is harder to fully align the mesogens with an increase in their dimensions. Notably, the measured enthalpy per gram of the sample is higher for 4ANTEM despite its lower epoxy equivalent coming from the same number of oxirane groups per mol of the compound and its greater molar mass compared to 3ANTEM.

The analysis of diaminodiphenylmethanes (DDMs) is more complex and complicated. The far greater heat of the reaction for 3ANTEM results in a far lower T_g_. The proposed explanation for this is that some of the curing enthalpy for 4ANTEM is hidden under the transition of the resin into the mesophase. Moreover, the curing of 4ANTEM is performed mostly in the LC state of the LCER, which is oriented at the molecular level. This causes the formation of a highly oriented polymer network, which acts similarly to crystals. The crystalline structure is much stiffer than the amorphous one, which may lead to an increase in T_g_ despite the 3ANTEM polymer network, which is cured with greater conversion of functional groups but in a chaotic, isotropic liquid state.

There is also an explanation for the effect of phenylenediamines (PDAs) on the T_g_ value. They are the smallest molecules among the used hardeners, and, as stated before, this allows them to penetrate the resin molecules more efficiently. This led to the greatest detected heat of reaction among the entire set of curing agents. The high extent of curing is the reason for the relatively high T_g_ of the networks formed with *p*-PDA. This isomer allows the achievement of a high T_g_ thanks to its structure and amine groups in the furthest possible position. There are significant difficulties in creating a network when epoxy groups are far from each other and additionally separated by halves of long, hydrocarbon spacers, so this process should be more possible with use of *p*-PDA as opposed *m*-PDA and *o*-PDA. The visible exception of this rule is the 3ANTEM/o-PDA system, wherein the highest curing enthalpy was detected. For some reason (probably related to the close proximity of amine groups), a reaction at low temperatures does not occur, but there is an abrupt start of the curing at over 200 °C, which can be correlated with the lower viscosity of the unreacted, hot mixture. This was also described in the previous section of this paper.

The last interesting conclusion can be drawn from the comparison of the 4,4′-DDM and 4,4′-ODA. The change of the methylene group to an oxygen atom (the corresponding structures are presented in the Figure 6) slightly increases the polarity of the molecule, making the whole polymer network stiffer due to the polar interactions between this oxygen atom and the ester and hydroxyl groups coming from the resin. This may result in a subtle increase in T_g_ with the use of 4,4’-ODA instead of 4,4′-DDM. Also, the interaction between ODAs and polar fragments of the mesogen can facilitate intercalation of the resin clusters and allow contact with reactive functional groups as a final effect of this process. This is evidenced by the higher curing enthalpies in the case of ODAs than in the case of DDMs.

## 4. Conclusions

Fourteen liquid-crystalline polymer networks were synthesised with a set of seven hardeners and two nonterminal LCERs. The effect of curing agent and resin structure was investigated in detail. The main and most important conclusions are listed below:

- Low-T_g_ LC polymer networks can be synthesized using the new kind of LCERs—nonterminal compounds;
- The introduction of the fourth aromatic ring into the mesogen core generally increases stiffness and the T_g_ of the network;
- The curing of LCERs can be constrained by the high viscosity of a mixture at low temperatures, especially when boosted by oligomer formation in the first minutes of the reaction;
- The larger core of resin facilitates intercalation and increases the extent of the cure;
- The prediction of the T_g_ of the polymers is possible but complicated, and the interpretation of the DSC curve in this regard is necessary;
- Polar interactions between resin and a curing agent may result in an increase in T_g_ thanks to the interactions themselves and easier penetration between resin scaffolds.

The aim of this paper was to assess and determine the effect of a nonterminal liquid crystalline epoxy resin structure and curing agent type on the glass transition of the polymer network. For these considerations, the heat of reaction conclusions and analyses are auxiliary but extremely helpful data. Overall, the reaction enthalpy is relatively small and generated in a very broad range of temperatures, so the reaction can be described as easy to control. This article provides a valuable screening method conducted via accounting for the thermal properties of novel, innovative polymer networks synthesised from a new class of monomers—nonterminal liquid crystalline epoxy resins. It can be a foundation for some more specific research concerning the chosen system for desired applications and properties.

## Figures and Tables

**Table 1:** Thermal and phase behaviour of monomers—collected data.

Transition Type	ΔH, kJ/mol	T_peak_, °C
3ANTEM		
Cr → Cr’	40.76	96.2
Cr’ → N	27.86	119.4
N → I	10.24	142.1
4ANTEM		
Cr → Cr’	33.97	102.8
Cr’ → SmA	12.55	133.8
SmA → N	8.53	144.4
N → I	9.32	213.7

**Table 2:** Curing enthalpy and glass transition temperature of investigated samples.

Curing Agent	3ANTEM		4ANTEM	
	ΔH, J/g	T_g_, °C	ΔH, J/g	T_g_, °C
4,4′-DDM	57.9	7.5	20.3	32.4
3,3′-DDM	40.5	5.5	17.6	30.8
o-PDA	166.1	37.0	110.2	12.8
m-PDA	37.7	−7.5	115.3	26.2
p-PDA	42.2	23.4	121.6	33.5
4,4′-ODA	75.4	32.0	87.7	32.4
3,4′-ODA	32.4	24.1	63.0	27.9

## Data Availability

Data are contained within the article.
